# Laparoscopic Sacropexy Versus Vaginal Sacrospinous Fixation for Pelvic Organ Prolapse: A Retrospective Comparison of Surgical Outcomes and Quality of Life

**DOI:** 10.3390/healthcare14111469

**Published:** 2026-05-26

**Authors:** Sima Ismayilova, Narmin Ismayilova, Jörg Engel, Anita Windhorst

**Affiliations:** 1 Department of Gynecology and Obstetrics, Verbundkrankenhaus Bernkastel, 54516 Wittlich, Germany; 2 Faculty of Medicine, Philipps-Universität, 35037 Marburg, Germany; ismayiln@students.uni-marburg.de; 3 Clinic Main-Spessart, 97816 Lohr am Main, Germany; joerg.engel@klinikum-msp.de; 4 Institute of Medical Informatics, Justus-Liebig-Universität, 35390 Gießen, Germany; anita.c.windhorst@informatik.med.uni-giessen.de

**Keywords:** pelvic organ prolapse, laparoscopic sacropexy, sacrospinous fixation, quality of life, surgical outcomes, urogynecology

## Abstract

**Highlights:**

**What are the main findings?**

- Both laparoscopic sacropexy and vaginal sacrospinous fixation improved quality of life across multiple domains with high patient satisfaction, but laparoscopic sacropexy showed zero intraoperative complications versus 17.6% with vaginal fixation (*p* = 0.02). A within-group improvement in sexual function scores was observed in LSC (*p* = 0.002); however, this finding was exploratory and not confirmed by significant between-group differences.
- Significant baseline differences between groups (higher ASA scores and comorbidities in the vaginal fixation group, all *p* < 0.05) reflect real-world selection bias, with higher-risk patients preferentially assigned to the vaginal approach to minimize physiological stress.

**What are the implications of the main findings?**

- Surgical approach selection should be individualized based on patient age, comorbidities, and sexual activity status, with laparoscopic sacropexy offering advantages for younger, healthier, sexually active women and vaginal sacrospinous fixation remaining valuable for high-risk patients.
- The observed selection bias highlights the need for prospective comparative studies with better-matched cohorts or propensity score analysis to definitively establish the independent effects of surgical technique on perioperative and sexual function outcomes.

**Abstract:**

**Background**: Pelvic organ prolapse (POP) significantly impacts women’s quality of life. Two established surgical approaches exist: laparoscopic sacropexy (LSC) and vaginal sacrospinous fixation (SSLF). This study compared surgical outcomes, complication rates, and quality of life between these techniques. **Methods**: This retrospective monocentric study included 58 patients treated between 2020 and 2023: 41 underwent LSC, and 17 underwent SSLF with vaginal hysterectomy. All procedures were performed by a single surgeon. Primary outcomes included operative time, complications, and hospital stay. Quality of life was assessed using the German Pelvic Floor Questionnaire (Deutscher Beckenboden-Fragebogen), King’s Health Questionnaire (KHQ), and patient satisfaction surveys. **Results**: Patient groups differed significantly in ASA scores (*p* = 0.023) and comorbidities, with SSLF patients showing higher morbidity. LSC demonstrated longer operative times (91 (75–115) vs. 73 (61–87) min, *p* = 0.05) but significantly fewer complications (0% vs. 17.6%, *p* = 0.02). Both methods showed significant improvements in bladder function, prolapse symptoms, and pelvic floor dysfunction scores (all *p* < 0.001). A within-group improvement in sexual function scores was observed in the LSC group (*p* = 0.002) but not in the SSLF group (*p* = 0.5); the between-group comparison of change scores was not significant (*p* = 0.8). No significant differences were found between groups regarding hospital stay duration or overall patient satisfaction (LSC: 95% vs. SSLF: 87% satisfied, *p* > 0.05). **Conclusions**: Both surgical approaches effectively treat POP with high patient satisfaction. LSC was associated with fewer observed complications and a within-group improvement in sexual function scores; SSLF was associated with shorter operative time and was applied in patients with higher morbidity. These associations may partly reflect baseline differences between groups and are considered hypothesis-generating. SSLF remains suitable for patients with higher morbidity when minimizing operative time and avoiding Trendelenburg positioning is advantageous.

## 1. Introduction

Pelvic organ prolapse (POP) is a common condition affecting women’s quality of life, with prevalence increasing with age. Approximately one-third of women experience some degree of pelvic organ descent, often accompanied by urinary or fecal incontinence [1,2]. The cumulative lifetime risk of undergoing prolapse surgery in the United States is approximately 11%, with over one-third requiring repeat procedures [3].

Conservative management options include pelvic floor muscle training and pessary use, but surgical intervention becomes necessary when symptoms significantly impair quality of life [4]. Multiple surgical techniques exist for POP correction, with laparoscopic sacropexy (LSC) and vaginal sacrospinous ligament fixation (SSLF) representing two widely accepted approaches in Germany.

Although both methods aim to restore anatomical support and improve symptoms, comparative data regarding patient-reported outcomes, particularly in sexually active women, remain limited. Previous studies comparing abdominal sacropexy with SSLF have shown differences in anatomical outcomes and dyspareunia rates [5], but direct comparisons between laparoscopic approaches and vaginal techniques performed by a single surgeon under standardized conditions are scarce.

This study exploratively compared LSC and SSLF regarding (1) perioperative outcomes, including operative time, complications, and hospital stay, (2) patient-reported quality-of-life improvements across multiple domains, and (3) patient satisfaction. Given the non-randomized retrospective design, findings are reported as descriptive associations intended to generate hypotheses for future prospective research.

## 2. Materials and Methods

### 2.1. Study Design and Ethical Approval

This retrospective monocentric study was conducted at Verbundkrankenhaus Bernkastel/Wittlich, Germany. Patients who underwent surgical treatment for pelvic organ prolapse between 2020 and 2023 were eligible for inclusion. The study was approved by the Ethics Committee of the State Medical Association of Rhineland-Palatinate in 2023 (approval number: 2023-17083). Eligible patients were contacted post-operatively, and written informed consent for retrospective data collection and analysis was obtained from all participants prior to study enrollment.

### 2.2. Patient Selection

All procedures were performed by a single experienced surgeon, ensuring standardization of surgical technique. Inclusion criteria were (1) diagnosis of symptomatic POP, (2) age 35 to 87 years, and (3) treatment with either LSC or SSLF. In the LSC group, the procedure consisted of laparoscopic sacropexy combined with laparoscopic supracervical hysterectomy with cervical preservation, or vault suspension in previously hysterectomized patients, without colporrhaphy. In the SSLF group, the procedure consisted of sacrospinous ligament fixation combined with vaginal hysterectomy and concomitant anterior and/or posterior colporrhaphy where indicated, or vault fixation in previously hysterectomized patients. Exclusion criteria included patients lacking decision-making capacity or the inability to provide informed consent.

Of 70 contacted patients, 12 declined participation, resulting in a final cohort of 58 patients: 41 in the LSC group, and 17 in the SSLF group. Surgical approach selection was based primarily on clinical decision-making considering patient comorbidities, with higher-risk patients (higher ASA scores, significant cardiovascular comorbidities) preferentially assigned to the vaginal approach to avoid prolonged Trendelenburg positioning and the physiological demands of laparoscopy.

### 2.3. Surgical Techniques

Laparoscopic sacropexy (LSC): The procedure involved standard laparoscopic port placement with the patient in the Trendelenburg position. In patients with previous hysterectomy (n = 9, 22%), the vaginal stump was identified and mobilized. In patients without previous hysterectomy (n = 32, 78%), laparoscopic supracervical hysterectomy was performed with cervical preservation and fixation. Following hysterectomy or stump identification, the vesicovaginal and rectovaginal spaces were dissected. A Y-shaped polypropylene mesh (Seratex^®^ B2 PA MR, SERAG-WIESSNER GmbH, 95119 Naila, Germany) was fixed to the anterior and posterior vaginal walls using absorbable tackers and/or non-absorbable sutures, then attached to the anterior longitudinal ligament at S1 level. The peritoneum was closed to extraperitonealize the mesh.

Vaginal sacrospinous ligament fixation (SSLF): Patients without previous hysterectomy (n = 13, 76%) underwent concurrent vaginal hysterectomy. Following hysterectomy or in patients with previous hysterectomy (n = 4, 24%), the sacrospinous ligament was identified, and the vaginal apex (or vaginal stump) was fixed using 2–3 permanent sutures placed approximately one fingerbreadth medial to the ischial spine to avoid neurovascular injury. Anterior and/or posterior colporrhaphy was performed as indicated.

Between the groups, differences in concomitant surgical procedures were present. In the vaginal group, anterior (n = 10) and posterior (n = 14) colporrhaphies were additionally performed, whereas no such procedures were carried out in the laparoscopic group. Furthermore, the type of hysterectomy differed between groups, with laparoscopic supracervical hysterectomy (LASH) performed exclusively in the laparoscopic group and vaginal hysterectomy exclusively in the vaginal group. These variables were considered potential confounders in the analysis.

Within the laparoscopic sacropexy (LSC) group, 32 of 41 patients (78%) underwent laparoscopic supracervical hysterectomy (LASH) with preservation of the cervix, while 9 patients (22%) had either a prior or concomitant hysterectomy. In contrast, in the sacrospinous ligament fixation (SSLF) group, 13 of 17 patients (76%) underwent a complete vaginal hysterectomy with removal of the cervix. Accordingly, the two groups differed substantially with regard to cervical status, with cervical preservation in the LSC group and cervical removal in the SSLF group.

### 2.4. Data Collection

Perioperative data were extracted from medical records, including patient demographics, obstetric history, ASA classification, comorbidities, operative time, perioperative hemoglobin changes (as a surrogate for estimated blood loss), intraoperative complications, and length of hospital stay.

Perioperative complications were identified through a retrospective review of patient medical records. Only intraoperative and immediate post-operative adverse events documented in the clinical records were included. No standardized classification system, such as the Clavien–Dindo classification, was applied for grading complications. Complication assessment was therefore based exclusively on routinely documented clinical events in the hospital records.

Quality of life was assessed using three validated questionnaires administered between April and August 2024. Patients were asked to retrospectively rate their symptoms both before surgery (pre-operative status) and at the time of survey completion (post-operative status), representing a median follow-up period of approximately 24–36 months after surgery.

1. German Pelvic Floor Questionnaire (Deutscher Beckenboden-Fragebogen) [6]: A 42-item validated instrument assessing four domains (bladder function, bowel function, prolapse symptoms, sexual function) with a total Pelvic Floor Dysfunction Score (maximum 40 points, higher scores indicating greater dysfunction).
2. King’s Health Questionnaire (KHQ) [7]: A disease-specific quality-of-life instrument for urinary incontinence comprising nine domains, scored 0–100 (higher scores indicating poorer quality of life).
3. Patient Satisfaction Questionnaire (ZUF-8) [8]: An 8-item instrument assessing treatment quality, fulfillment of expectations, and willingness to recommend or repeat the procedure.

Sexual activity was categorized based on patient-reported information into three groups: “none”, “occasional”, and “regular” sexual activity. This classification was not based on a validated or standardized instrument but derived from routine clinical history documentation.

### 2.5. Statistical Analysis

Statistical analysis was performed using IBM SPSS Statistics (Version 29.0, IBM Corporation, Armonk, NY, USA) and R (Version 4.3.2, R Foundation for Statistical Computing, Vienna, Austria) [9] with the packages Hmisc (Version 5.1-1) [10] and packages from the tidyverse (Version 2.0.0) [11]. Descriptive statistics included medians with interquartile ranges (25th–75th percentile) for continuous variables and frequencies with percentages for categorical variables.

Between-group comparisons utilized Mann–Whitney U tests for continuous variables and Fisher’s exact tests for categorical data. For categorical variables with multiple categories, Fisher’s exact test with simulated *p*-values (based on 2000 replications) was employed. Pre- and post-operative comparisons employed Wilcoxon signed-rank tests for paired data. Statistical significance was defined as *p* ≤ 0.05 (two-sided).

Given the use of non-parametric statistical methods (Mann–Whitney U test, Wilcoxon signed-rank test), which are based on rank ordering rather than assumptions about the distribution of means, continuous data are presented as median (25th–75th percentile) throughout. This approach is statistically consistent with the analytical methods and more appropriate for ordinal questionnaire data and modest sample sizes for the sexual function subscale. The high proportion of sexually inactive patients at baseline (LSC: 51%; SSLF: 35%) resulted in a substantial mass of zero scores. Within-group comparisons (Wilcoxon signed-rank test) were therefore restricted to patients with scorable responses at both timepoints, while between-group comparisons of change scores (Mann–Whitney U test) were performed on all available data. As these two analyses operate on different patient populations, their *p*-values are not directly comparable and should be interpreted independently. All sexual function results should be interpreted with this constraint in mind.

Missing data were handled using available case analysis, with sample sizes reported for each analysis. Given the exploratory nature of this study, no adjustment for multiplicity (e.g., Bonferroni correction or false discovery rate control) was applied. The large number of outcome comparisons across questionnaire domains reflects the multidimensional assessment of pelvic floor dysfunction rather than a confirmatory testing strategy; accordingly, all reported *p*-values should be interpreted as descriptive and hypothesis-generating, not as evidence of effect in the inferential sense. Results with *p* ≤ 0.05 indicate statistical signals warranting further investigation in adequately powered prospective studies.

## 3. Results

### 3.1. Patient Characteristics

The study included 58 patients: 41 in the LSC group and 17 in the SSLF group. Baseline characteristics are presented in Table 1. Groups did not differ regarding age (LSC: median 63 years, IQR 58–70 vs. SSLF: median 65 years, IQR 57–72, *p* = 0.61), BMI (27, IQR 24–29 vs. 27, IQR 23–30, *p* = 1.0), or postmenopausal status (85% vs. 94%, *p* = 0.72).

However, differences were observed in parity and morbidity. SSLF patients had higher birth rates (median 2.0 vs. 2.0, but different distributions, *p* = 0.04) and higher ASA scores (*p* = 0.023), with 41% having ASA-III classification versus 12% in the LSC group. SSLF patients also had more pre-existing conditions, particularly arterial hypertension (71% vs. 32%, *p* = 0.009) and diabetes mellitus (29% vs. 5%, *p* = 0.02).

Previous hysterectomy was present in 22% of LSC and 24% of SSLF patients (*p* = 1.0).

### 3.2. Perioperative Outcomes

LSC procedures were associated with longer operative time compared with SSLF (91 (75–115) vs. 73 (61–87) min, *p* = 0.05), though the absolute difference was only 14 min (Table 2).

Perioperative hemoglobin changes were modest in both groups. Pre-operative hemoglobin was similar (LSC: median 13.7 g/dL, IQR 13.3–14.2 vs. SSLF: median 13.6 g/dL, IQR 12.5–13.9, *p* = 0.3), with post-operative values of median 13 (12–13) and 12 (11–12) g/dL, respectively (*p* = 0.01).

Hospital stay duration did not differ significantly between groups (LSC: 3.0 (3.0–3.0) vs. SSLF: 3.0 (3.0–3.0) days, *p* = 0.5).

A difference in observed complication rates was noted between groups (*p* = 0.02). The LSC group had no intraoperative complications, while the SSLF group experienced three complications (17.6%): one bladder injury, one rectal injury, and one intraoperative bradycardia requiring cardiac massage.

### 3.3. Quality-of-Life Outcomes

#### 3.3.1. German Pelvic Floor Questionnaire

Within-group improvements reaching the pre-specified threshold of *p* ≤ 0.05 were observed across multiple domains in both groups (Table 3). In the overall cohort, bladder function scores improved from a median of 4.0 (IQR 3.0–5.0) pre-operatively to 2.0 (IQR 1.0–3.0) post-operatively (*p* < 0.001). Prolapse symptom scores decreased from 4.7 (IQR 3.3–6.0) to 0.7 (IQR 0.0–2.7, *p* < 0.001), and the total pelvic floor dysfunction score declined from 12.0 (IQR 10.0–15.0) to 5.0 (IQR 3.0–10.0, *p* < 0.001). Sexual function scores also improved from 0.5 (IQR 0.0–3.3) to 0.0 (IQR 0.0–1.0, *p* = 0.005), while bowel function showed modest improvement from 2.1 (IQR 0.9–2.9) to 1.8 (IQR 0.6–2.6, *p* = 0.05).

When analyzed by procedure type, within the LSC group, improvements were observed in bladder function (median change 0.0, IQR −0.4 to 0.0, *p* < 0.001), prolapse symptoms (median change −1.6, IQR −3.2 to 0.0, *p* < 0.001), sexual function (median change 0.0, IQR −1.0 to 0.0, *p* = 0.002), and overall pelvic floor dysfunction (median change −6.6, IQR −8.4 to −0.2, *p* < 0.001). Bowel function did not improve significantly (*p* = 0.3).

Within the SSLF group, improvements in bladder function (median change 0.0, IQR −1.2 to 0.0, *p* = 0.007), prolapse symptoms (median change −1.6, IQR −3.8 to −0.7, *p* = 0.008), and overall pelvic floor dysfunction (median change −5.8, IQR −10.8 to −3.6, *p* = 0.006) were observed. However, sexual function (median change 0.0, IQR −1.9 to 0.0, *p* = 0.5) and bowel function (*p* = 0.2) did not improve in the SSLF group.

Direct comparison of change scores between surgical approaches revealed no statistically significant differences in any domain (bladder function *p* = 0.3, bowel function *p* = 0.3, prolapse symptoms *p* = 0.6, sexual function *p* = 0.8, total PFD score *p* = 0.4), though this may reflect limited statistical power due to the smaller SSLF group.

#### 3.3.2. King’s Health Questionnaire

The KHQ demonstrated comprehensive quality-of-life improvements in the LSC group across all ten domains (Table 4). Improvements were observed in general health perception (median change 0, IQR −25 to 0, *p* < 0.001), incontinence impact (median change −33, IQR −67 to 0, *p* < 0.001), role limitations (median change −33, IQR −67 to 0, *p* < 0.001), physical limitations (median change −33, IQR −67 to 0, *p* < 0.001), social limitations (median change −28, IQR −33 to 0, *p* < 0.001), personal relationships (median change −33, IQR −50 to 0, *p* < 0.001), emotions (median change −33, IQR −36 to 0, *p* < 0.001), sleep/energy (median change −25, IQR −33 to 0, *p* < 0.001), severity measures (median change −7, IQR −33 to 0, *p* = 0.002), overactive bladder symptoms (median change −6, IQR −11 to −1, *p* < 0.001), and symptom severity scale (median change −17, IQR −56 to 0, *p* < 0.001).

In the SSLF group, improvements occurred in six domains: general health perception (median change −25, IQR −50 to 0, *p* = 0.001), incontinence impact (median change −33, IQR −67 to 0, *p* < 0.001), role limitations (median change −33, IQR −50 to 0, *p* < 0.001), physical limitations (median change −33, IQR −75 to −8, *p* = 0.008), social limitations (median change −22, IQR −44 to 0, *p* = 0.04), and symptom severity scale (median change −44, IQR −75 to −9, *p* = 0.006). However, improvements in personal relationships (*p* = 0.3), emotions (*p* = 0.07), sleep/energy (*p* = 0.06), and severity measures (*p* = 0.1) did not reach statistical significance.

Direct comparison between groups showed no differences in the magnitude of improvement for any KHQ domain (all *p* > 0.05), with *p*-values ranging from 0.2 (symptom severity scale) to 1.0 (severity measures).

### 3.4. Patient Satisfaction

**Figure 1:**
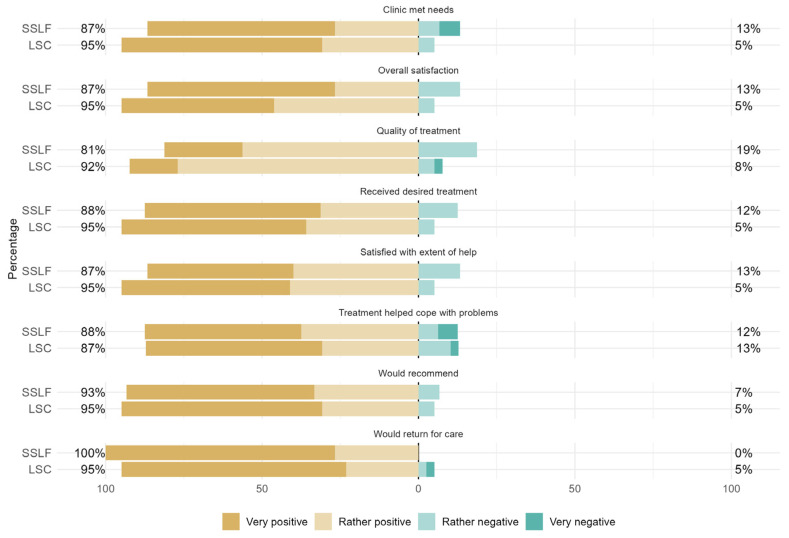
Patient satisfaction by surgical approach. Distribution of patient satisfaction responses across eight domains, comparing laparoscopic sacropexy (LSC, n = 39) and sacrospinous ligament fixation (SSLF, n = 15). Responses are categorized as very positive (dark gold), rather positive (light gold), rather negative (light teal), and very negative (dark teal). Percentages on the left represent combined positive responses; percentages on the right represent combined negative responses. No statistically significant differences were observed between groups for any satisfaction domain (all *p* ≥ 0.3, Fisher’s exact test with simulated *p*-values based on 2000 replications). Sample sizes vary slightly by question due to missing responses; ranges: LSC 37-39, SSLF 13-15.

Patient satisfaction was uniformly high across both surgical approaches (Figure 1, Table 5). Overall satisfaction was reported by 95% of LSC patients and 87% of SSLF patients (*p* = 0.32). When rating treatment quality, 92% of LSC patients versus 87% of SSLF patients provided positive ratings (excellent or good, *p* = 0.32). Nearly all patients in both groups reported receiving their desired treatment (LSC: 95%, SSLF: 93%, *p* = 0.62) and would recommend the procedure to a friend (LSC: 95%, SSLF: 93%, *p* = 1.0).

Willingness to return for care if needed was particularly high, with 95% of LSC patients and 100% of SSLF patients responding affirmatively (*p* = 1.0). No statistically significant differences in any satisfaction domain were observed between surgical approaches; this finding is consistent with comparable patient acceptance of both techniques, though the study was not powered to detect small differences in satisfaction.

Among sexually active patients, new-onset dyspareunia was reported by 1 of 20 LSC patients (5%) and 3 of 11 SSLF patients (27%, *p* = 0.09), though this difference did not reach statistical significance.

## 4. Discussion

This study compared two established surgical approaches for POP treatment, performed by a single surgeon under standardized conditions. Both LSC and SSLF were associated with improvements in quality of life across multiple domains and high patient satisfaction. Differences were observed in complication rates, operative time, and sexual function scores.

### 4.1. Patient Selection and Baseline Characteristics

The significant baseline differences between groups (ASA classification *p* = 0.023, arterial hypertension *p* = 0.009, diabetes mellitus *p* = 0.02, absence of comorbidities *p* < 0.001) strongly suggest systematic selection bias rather than random allocation. This confounding by indication is a fundamental limitation of the retrospective design: patients with higher morbidity were preferentially assigned to SSLF to avoid prolonged Trendelenburg positioning and the physiological demands of laparoscopy, while healthier patients received LSC.

This pragmatic patient selection mirrors real-world clinical practice and suggests that SSLF remains valuable for high-risk patients where minimizing physiological stress is paramount. However, the lower complication rate observed in the LSC group may partly or entirely reflect healthier baseline status rather than a technique-specific effect; no causal attribution is possible from these data. Propensity score matching or multivariable adjustment would be required to isolate the independent effect of surgical technique, though our sample size precludes such analysis [12]. Specifically, with only 17 patients in the SSLF group and three observed complications, the events-per-variable ratio falls well below the commonly recommended threshold of ten events per predictor variable for stable logistic regression estimates; propensity score models would face analogous instability. All observed between-group differences should therefore be read as unadjusted descriptive associations, with residual confounding by indication remaining a plausible alternative explanation for any apparent effect.

The higher parity in the SSLF group (*p* = 0.04) may also reflect the selection of patients with greater pelvic floor damage from multiple vaginal deliveries, potentially indicating more severe prolapse or different anatomical considerations influencing surgical approach selection.

### 4.2. Perioperative Outcomes

The 14 min difference in median operative time (LSC: 91 min vs. SSLF: 73 min, *p* = 0.05), while statistically significant, has limited clinical relevance. Previous meta-analyses comparing abdominal sacropexy with SSLF reported weighted mean differences of approximately 25 min favoring SSLF [5]. Our more modest difference may partly reflect the efficiency of contemporary laparoscopic techniques, including the use of tacking devices for mesh fixation and direct suture placement under vision during SSLF.

A difference in observed complication rates was noted between groups: no complications in LSC versus three events (17.6%) in SSLF (*p* = 0.02), two of which were directly procedure-related. However, this finding warrants cautious interpretation. Reporting zero complications in 41 laparoscopic procedures is unusually favorable. This rate may reflect conservative complication ascertainment, the absence of systematic Clavien–Dindo grading, single-surgeon expertise, or chance variation in a modest sample. It should be acknowledged that no systematic Clavien–Dindo classification was applied and that complications were assessed retrospectively based solely on documentation in the medical records. Therefore, the observed 0% complication rate in the laparoscopic (LSC) group may represent an underestimation of the true complication rate, as minor or undocumented events may not have been captured. The bladder and rectal injuries in the SSLF group occurred in patients undergoing surgery with altered anatomy from previous pelvic procedures, highlighting anatomical challenges in the vaginal approach. This pattern is broadly consistent with meta-analytic evidence reporting lower complication rates with sacropexy approaches [5,13], though differences in patient selection and complication definition limit direct comparability. The intraoperative bradycardia requiring cardiac massage in one SSLF patient underscores the potential for unexpected cardiovascular events even with regional anesthesia and the vaginal approach.

Hemoglobin changes were modest in both groups, with slightly greater decreases in SSLF patients (median change −1.2 g/dL vs. −0.7 g/dL, *p* = 0.15). The lack of systematic documentation of estimated blood loss represents a limitation, as hemoglobin change is an imperfect surrogate influenced by fluid administration and hemodilution. Hospital stay duration did not differ between groups (median 3.0 days for both, *p* = 0.5).

### 4.3. Quality-of-Life Improvements

Both surgical approaches were associated with improvements across multiple quality-of-life domains. The improvements in bladder function and prolapse-specific symptoms were comparable between techniques, with both groups showing median reductions in prolapse symptom scores of approximately 1.6 points on a 10-point scale (LSC: median change −1.6, IQR −3.2 to 0.0, *p* < 0.001; SSLF: median change −1.6, IQR −3.8 to −0.7, *p* = 0.008). This pattern is consistent with comparable anatomical restoration of pelvic support across surgical routes, though this cannot be confirmed from patient-reported data alone.

A differential pattern in sexual function scores was observed between groups; given the exploratory design and the analytic constraints described in Section 2.5, this pattern should not be interpreted as evidence of a differential treatment effect. LSC showed an improvement in sexual function scores (median change 0.0, IQR −1.0 to 0.0, *p* = 0.002), while SSLF did not (median change 0.0, IQR −1.9 to 0.0, *p* = 0.5). Among sexually active patients, new-onset dyspareunia was more common after SSLF (3/11, 27.3%) compared with LSC (1/20, 5.0%, *p* = 0.09), though this difference did not reach statistical significance in our sample size.

This pattern may reflect multiple anatomical factors. The sacropexy approach maintains the physiological vaginal axis along the line of the sacrum, potentially reducing mechanical stress during intercourse. Historical data from open abdominal approaches documented mean vaginal lengths of 11.3 cm after sacropexy versus 8.2 cm after SSLF [14]. Whether anatomical findings from open abdominal procedures reported in 1993 are applicable to contemporary laparoscopic techniques is uncertain; vaginal dimensions were not measured in the present study, and extrapolation is not warranted. The observed pattern, a within-group improvement in LSC but not in SSLF, alongside a non-significant between-group comparison (*p* = 0.8), is consistent with the hypothesis that surgical approach may influence sexual function outcomes; this hypothesis requires testing in adequately powered prospective studies before informing clinical counseling.

Improvements were observed across all ten KHQ domains in the LSC group and six of ten in the SSLF group; this difference in pattern may reflect the greater baseline morbidity of SSLF patients rather than a technique-specific effect. However, the lack of statistically significant differences in change scores between groups (all *p* > 0.05) precludes conclusions about differential efficacy and may reflect Type II error given the modest sample size. The SSLF group’s baseline higher morbidity may have influenced their capacity for improvement in domains such as sleep/energy and emotions, which may be more strongly influenced by general health status than by prolapse-specific factors.

Notably, bowel function did not improve significantly in either group (LSC *p* = 0.3, SSLF *p* = 0.2), consistent with the possibility that bowel symptoms reflected functional rather than anatomical prolapse-related factors.

### 4.4. Clinical Implications

The observed associations in this exploratory cohort are consistent with an individualized approach to surgical selection for POP. In this cohort, LSC was associated with fewer observed complications and a within-group improvement in sexual function scores; whether these reflect technique-specific effects or patient selection cannot be determined from these data. Whether preservation of the vaginal axis with sacropexy contributes to the observed sexual function pattern remains to be established.

For patients with significant comorbidities (ASA-III, multiple medical conditions), SSLF was associated with effective prolapse treatment while avoiding prolonged Trendelenburg positioning and the physiological demands of laparoscopy. The shorter operative time, while modest, may benefit high-risk patients, and the vaginal approach avoids intra-abdominal mesh placement with its attendant long-term risks.

The decision should incorporate comorbidity profiles, sexual activity status, patient preferences regarding mesh use, and surgical expertise. The present cohort illustrates that comorbidity-based patient selection influences which technique is applied; whether this optimizes outcomes cannot be determined from an uncontrolled observational design.

### 4.5. Comparison with Existing Literature

The descriptive patterns observed in this cohort are broadly consistent with existing meta-analyses, though differences in surgical approach, patient selection, and study design limit direct comparability. Zhang et al. [5] reported that sacropexy demonstrated higher anatomical success rates (91.45% vs. 88.32%), lower recurrence rates (8.32% vs. 11.58%), and lower dyspareunia rates (4.67% vs. 14.36%) compared with SSLF, while SSLF showed advantages in operative time, bleeding, wound infections, and gastrointestinal complications.

A key distinction in our study is the laparoscopic rather than open abdominal approach, which may partly account for the favorable complication profile observed in this cohort compared with meta-analysis data that predominantly included open procedures. Contemporary laparoscopic techniques have evolved to minimize the bleeding and infection risks associated with laparotomy while preserving the anatomical benefits of sacropexy.

Sarlos et al. [15] demonstrated sustained quality-of-life improvements following laparoscopic sacropexy with extended follow-up, consistent with the durability of within-group improvements observed in the present cohort. Our finding of similar overall satisfaction between approaches (LSC: 95% vs. SSLF: 87%, *p* = 0.32) despite technical differences is consistent with both techniques meeting patient expectations when applied by an experienced surgeon, though the small SSLF group and non-significant difference preclude firm conclusions.

### 4.6. Strengths and Limitations

Important strengths of this study include standardization of technique with all procedures performed by a single experienced surgeon, ensuring technical consistency throughout the study period (2020–2023). The comprehensive quality-of-life assessment using multiple validated instruments (German Pelvic Floor Questionnaire [6], King’s Health Questionnaire [7], and patient satisfaction surveys [8]) provides multidimensional evaluation of both objective and patient-reported outcomes.

However, several important limitations warrant acknowledgment. The retrospective design introduces inherent biases, particularly selection bias, as discussed in Section 4.1. Clinical decision-making based on comorbidity profiles resulted in significant baseline differences between groups (ASA classification *p* = 0.023, arterial hypertension *p* = 0.009, diabetes mellitus *p* = 0.02, absence of comorbidities *p* < 0.001). This confounding by indication limits our ability to isolate the independent effect of surgical technique from patient-level factors. The lower complication rate observed in the LSC group may partly reflect a healthier baseline status rather than a technique-specific effect. Propensity score matching or multivariable adjustment would be required to control for these differences [12], though our sample size precludes such analysis.

Two further sources of uncontrolled confounding relate to procedural heterogeneity between groups. Anterior and posterior colporrhaphies were performed exclusively in the SSLF group; these additional repairs may independently influence functional outcomes and operative time. Hysterectomy type also differed systematically: 78% of LSC patients underwent LASH with cervical preservation, whereas 76% of SSLF patients underwent total vaginal hysterectomy with cervical removal. Cervical preservation versus excision may influence vaginal anatomy and sexual function independently of the apical suspension technique. Neither of these procedural differences was adjusted for in any analysis.

The relatively small sample size, particularly in the SSLF group (n = 17), limits statistical power to detect clinically meaningful differences in secondary outcomes. The lack of statistically significant differences in quality-of-life change scores between groups (all *p* > 0.05) may reflect Type II error rather than true equivalence. Post hoc power calculations were performed to quantify this uncertainty. For the complication comparison (0% vs. 17.6%, Fisher’s exact test, n = 41 vs. 17, two-sided α = 0.05), achieved power was approximately 61% using a continuity-corrected effect size estimate. For the between-group comparison of sexual function change scores (Mann–Whitney U test, n = 40 vs. 17), power to detect a medium effect (r = 0.3) was 53%; only effects of d ≥ 0.83 would have been detectable at 80% power. Non-significant between-group results should therefore be interpreted as inconclusive rather than as evidence of equivalence.

The retrospective assessment of pre-operative and post-operative symptoms represents a central methodological limitation. Patients were surveyed in 2024 (April–August) and asked to recall their symptom severity both before surgery (2020–2023) and at the time of survey completion, rather than completing questionnaires prospectively at these timepoints. This introduces recall bias, as patients’ current symptom status may influence their recollection of pre-operative severity. Response shift compounds this problem. Following successful surgery, patients tend to rate their pre-operative state more negatively in retrospect than they would have at the time, because their internal frame of reference has changed [16]. Both mechanisms systematically inflate observed pre–post effect sizes. The within-group improvements reported in this study should therefore be interpreted as upper-bound estimates of the true treatment effect rather than precise measures of change. Prospective administration of validated questionnaires at baseline and defined follow-up intervals would have been methodologically superior and is recommended for future studies.

The follow-up duration (median approximately 24–36 months based on surgery dates 2020–2023 and survey timing in 2024) may be insufficient to detect late mesh-related complications. Mesh erosion following sacropexy can manifest beyond 36 months [17], and complications assessed through patient-reported outcomes alone may miss asymptomatic events. Studies with at least 5 years of follow-up and systematic clinical examination are needed to assess long-term safety.

Systematic POP-Q staging was not available in the dataset and could not be retrospectively reconstructed from operative reports or patient records. Prolapse severity was therefore not uniformly documented in a standardized manner, which limits comparability with published series and precludes objective anatomical outcome assessment. Vaginal length measurements were not obtained, limiting objective evaluation of the sexual function findings. Future prospective studies should include standardized POP-Q staging and validated sexual function assessment using the FSFI.

The sexual activity categorization used (none, occasional, regular) lacks standardized frequency definitions, which limits comparability with other studies. The single-surgeon, monocentric design, while ensuring technical consistency, limits generalizability to settings with multiple surgeons or different patient populations.

Future research should prioritize prospective designs with adequate statistical power, baseline and interval quality-of-life assessment using validated instruments, standardized anatomical evaluation with POP-Q, and systematic complication recording according to the Clavien–Dindo classification. Validated sexual function assessment using the FSFI, follow-up of at least 5 years for mesh-related safety, and stratification or matching on baseline comorbidity are essential to isolate technique-specific effects.

This study did not assess objective anatomical outcomes at follow-up, including post-treatment POP-Q staging, anatomical recurrence rates, need for revision surgery, or mesh-related complications identified through systematic clinical examination. Future prospective studies should include structured follow-up protocols addressing these endpoints.

### 4.7. Emerging Techniques

Newer approaches such as laparoscopic lateral suspension (LLS) and robotic-assisted sacropexy represent evolving alternatives that may offer additional benefits. LLS utilizes bilateral fixation to the pelvic sidewalls, potentially providing more physiological support while avoiding dissection near the promontory, autonomic nerves, and major vessels [18]. Robotic assistance may facilitate precise mesh placement and intracorporeal suturing, though cost considerations remain relevant [19,20,21].

Bilateral sacrospinous fixation using specialized instruments represents another innovation, potentially combining the physiological axis restoration of bilateral fixation with the efficiency of the vaginal approach, though larger studies are needed to establish its role.

## 5. Conclusions

Both laparoscopic sacropexy and vaginal sacrospinous fixation were associated with substantial improvements in quality of life across multiple domains and high patient satisfaction in this exploratory cohort study. LSC was associated with fewer observed intraoperative complications and a within-group improvement in sexual function scores; SSLF was associated with shorter operative time and was applied preferentially in patients with higher medical comorbidity. These associations are unadjusted and may partly reflect systematic differences in patient selection rather than technique-specific effects.

The observed pattern is consistent with the hypothesis that surgical approach, patient morbidity profile, and sexual function outcomes are interrelated in the treatment of pelvic organ prolapse. Prospective studies with standardized outcome assessment, adequate sample sizes, and pre-specified adjustment for confounders are required to test this hypothesis and to determine whether the observed differences persist after accounting for baseline imbalances. These findings underscore the clinical relevance of maintaining expertise in both approaches to allow individualized patient selection for women with pelvic organ prolapse and provide a descriptive basis for the design of future comparative research.

## Figures and Tables

**Table 1:** Patient demographics and clinical characteristics.

Characteristic	LSC (n = 41)	SSLF (n = 17)	Total (n = 58)	*p*-Value
**Demographics**				
Age (years)	63 (58–70)	65 (57–72)	64 (57–70)	0.61
Postmenopausal, n (%)	35 (85.4)	16 (94.1)	51 (87.9)	0.72
Body mass index (kg/m^2^)	27 (24–29)	27 (23–30)	27 (23–30)	1.0
BMI > 25, n (%)	25 (61.0)	11 (64.7)	36 (62.1)	1.0
BMI > 30, n (%)	9 (22.0)	4 (23.5)	13 (22.4)	1.0
Current smoker, n (%)	2/35 (5.7)	2/15 (13.3)	4/50 (8.0)	0.62
**Obstetric History**				
Parity	2.0 (1.0–2.0)	2.0 (2.0–3.0)	2.0 (1.0–3.0)	0.04 *
Number of births, n (%)				0.04 *
0	1 (2.4)	1 (5.9)	2 (3.4)	
1	13 (31.7)	1 (5.9)	14 (24.1)	
2	18 (43.9)	8 (47.1)	26 (44.8)	
3	8 (19.5)	3 (17.6)	11 (19.0)	
4	1 (2.4)	3 (17.6)	4 (6.9)	
5	0 (0)	1 (5.9)	1 (1.7)	
Spontaneous vaginal delivery	2.0 (1.0–2.0)	2.0 (2.0–3.0)	2.0 (1.0–2.8)	0.06
≥1 SVD, n (%)	38 (92.7)	16 (94.1)	54 (93.1)	1.0
Vacuum extraction, n (%)	3 (7.3)	0 (0)	3 (5.2)	0.52
Cesarean section, n (%)	2 (4.9)	3 (17.6)	5 (8.6)	0.12
**Medical History**				
Previous hysterectomy, n (%)	9 (22.0)	4 (23.5)	13 (22.4)	1.0
ASA classification, n (%)				0.02 *
ASA-I	6 (14.6)	0 (0)	6 (10.3)	
ASA-II	30 (73.2)	10 (58.8)	40 (69.0)	
ASA-III	5 (12.2)	7 (41.2)	12 (20.7)	
**Comorbidities, n (%)**				
No comorbidities	17/39 (43.6)	0/16 (0)	17/55 (30.9)	<0.001 *
Arterial hypertension	13 (31.7)	12 (70.6)	25 (43.1)	0.009 *
Diabetes mellitus	2 (4.9)	5 (29.4)	7 (12.1)	0.02 *
Depression	2 (4.9)	1 (5.9)	3 (5.2)	1.0
Hypothyroidism	2 (4.9)	1 (5.9)	3 (5.2)	1.0
Pulmonary embolism (history)	2 (4.9)	0 (0)	2 (3.4)	1.0
Breast cancer (history)	1 (2.4)	2 (11.8)	3 (5.2)	0.21
Asthma	3 (7.3)	0 (0)	3 (5.2)	0.51
Cardiac arrhythmia	0 (0)	1 (5.9)	1 (1.7)	0.31
Coronary heart disease	1 (2.4)	0 (0)	1 (1.7)	1.0
**Previous Pelvic Surgery, n (%)**				
Cholecystectomy	2 (4.9)	4 (23.5)	6 (10.3)	0.06
Adnexectomy	5 (12.2)	2 (11.8)	7 (12.1)	1.0
Appendectomy	1 (2.4)	1 (5.9)	2 (3.4)	0.51
Thyroidectomy	5 (12.2)	1 (5.9)	6 (10.3)	0.71

Data are presented as median (25th–75th percentile) for continuous variables and number (percentage) for categorical variables. Abbreviations: LSC = laparoscopic sacropexy; SSLF = sacrospinous ligament fixation; BMI = body mass index; SVD = spontaneous vaginal delivery; ASA = American Society of Anesthesiologists. Statistical tests: Mann–Whitney U test for continuous variables; Fisher’s exact test for categorical variables (with simulated *p*-values based on 2000 replications for multi-category variables). * Statistically significant.

**Table 2:** Perioperative outcomes.

Parameter	LSC (n = 41)	SSLF (n = 17)	Total (n = 58)	*p*-Value
**Operative Data**				
Operative time (min)	91 (75–115)	73 (61–87)	86 (69–114)	0.05
**Hemoglobin Levels (g/dL)**				
Pre-operative	13.7 (13.3–14.2)	13.6 (12.5–13.9)	13.6 (13.2–14.0)	0.3
Post-operative	13 (12–13)	12 (11–12)	12 (12–13)	0.01
Hemoglobin change	−0.7 (−1.5 to 0.0)	−1.2 (−2.0 to −0.5)	−1.0 (−1.6 to −0.2)	0.15
**Post-Operative Course**				
Hospital stay (days)	3.0 (3.0–3.0)	3.0 (3.0–3.0)	3.0 (3.0–3.0)	0.5
**Complications, n (%)**				
Overall complications	0 (0)	3 (17.6)	3 (5.2)	0.02 *
**Intraoperative Complications, n (%)**				
Bladder injury	0 (0)	1 (5.9)	1 (1.7)	0.29
Rectal injury	0 (0)	1 (5.9)	1 (1.7)	0.29
Bradycardia requiring cardiac massage	0 (0)	1 (5.9)	1 (1.7)	0.29
**Sexual Activity**				
Pre-operative, n (%)				0.1
None	21 (51.2)	6 (35.3)	27 (46.6)	
Regular	8 (19.5)	8 (47.1)	16 (27.6)	
Occasional	12 (29.3)	3 (17.6)	15 (25.9)	
Post-operative, n (%)				0.4
None	21 (51.2)	6 (35.3)	27 (46.6)	
Regular	7 (17.1)	5 (29.4)	12 (20.7)	
Occasional	13 (31.7)	6 (35.3)	19 (32.8)	
**New-Onset Dyspareunia, n (%)**	1/20 (5.0)	3/11 (27.3)	4/31 (12.9)	0.09
**Colporrhaphy**				
Anterior	0 (0)	10 (58.8%)	10 (17.2)	*p* < 0.001
Posterior	0 (0)	14 (82.5%)	14 (24.4)	*p* < 0.001
**Hysterectomy**				
LASH	32 (78.5)	0 (0)	32 (55.2)	*p* < 0.001
Vaginal	0 (0)	13 (76.5%)	13 (22.4)	*p* < 0.001

Data are presented as median (25th–75th percentile) for continuous variables and number (percentage) for categorical variables. LSC = laparoscopic sacropexy; SSLF = sacrospinous ligament fixation; LASH = Laparoscopic Supracervical Hysterectomy. Statistical tests: Mann–Whitney U test for continuous variables; Fisher’s exact test for categorical variables. * Statistically significant.

**Table 3:** German Pelvic Floor Questionnaire Scores (higher scores = higher dysfunction).

Domain (Range)	Time	LSC (n = 40)	SSLF (n = 17)	Total (n = 57)	*p*-Value
**Bladder Function (0–10)**					
	Pre-op	4 (3–5)	4 (4–6)	4 (3–5)	
	Post-op	2 (1–3)	2 (1–3)	2 (1–3)	
	Change	0.0 (−0.4 to 0.0)	0.0 (−1.2 to 0.0)	0.0 (−0.6 to 0.0)	0.3 ^†^
	*p*-value *	<0.001	0.007	<0.001	
**Bowel Function (0–10)**					
	Pre-op	2.1 (0.9–2.7)	1.8 (0.9–2.9)	2.1 (0.9–2.9)	
	Post-op	1.8 (0.8–2.6)	1.8 (0.6–2.4)	1.8 (0.6–2.6)	
	Change	−3 (−5 to 0)	−5 (−6 to −1)	−3 (−5 to 0)	0.3 ^†^
	*p*-value *	0.3	0.2	0.05	
**Prolapse Symptoms (0–10)**					
	Pre-op	3.7 (2.7–6.0)	5 (5–6)	4.7 (3.3–6.0)	
	Post-op	0.7 (0.0–2.7)	0 (0–5)	0.7 (0.0–2.7)	
	Change	−1.6 (−3.2 to 0.0)	−1.6 (−3.8 to −0.7)	−1.6 (−3.3 to −0.2)	0.6 ^†^
	*p*-value *	<0.001	0.008	<0.001	
**Sexual Function (0–10)**					
	Pre-op	0.5 (0.0–3.3)	0 (0–2)	0.5 (0.0–3.3)	
	Post-op	0.0 (0.0–1.0)	0 (0–1)	0.0 (0.0–1.0)	
	Change	0.0 (−1.0 to 0.0)	0.0 (−1.9 to 0.0)	0.0 (−1.0 to 0.0)	0.8 ^†^
	*p*-value *	0.002	0.5	0.005	
**Total PFD Score (0–40)**					
	Pre-op	12 (10–15)	13 (10–17)	12 (10–15)	
	Post-op	5 (4–10)	5 (3–10)	5 (3–10)	
	Change	−6.6 (−8.4 to −0.2)	−5.8 (−10.8 to −3.6)	−6.4 (−8.6 to −0.7)	0.4 ^†^
	*p*-value *	<0.001	0.006	<0.001	

Data are presented as median (25th–75th percentile). Higher scores indicate greater dysfunction. LSC = laparoscopic sacropexy; SSLF = sacrospinous ligament fixation; PFD = pelvic floor dysfunction. * Wilcoxon signed-rank test for paired comparisons (pre- vs. post-operative within group). ^†^ Mann–Whitney U test for between-group comparison of change scores. Change scores represent the median of individual paired differences (post-operative minus pre-operative) at patient level, which may differ from the arithmetic difference between-group medians. A statistically significant result can occur even when the median paired difference is 0.0, if the distribution of non-zero differences is systematically shifted in one direction. Note that bowel function change scores are reported on the raw item scale and may therefore appear numerically discordant with domain-level group medians.

**Table 4:** Outcome King’s Health Questionnaire.

Domain (0–100)	Time	LSC (n = 41)	SSLF (n = 17)	Total (n = 58)	*p*-Value
**General Health Perception**					
	Pre-op	50 (25–50)	50 (50–75)	50 (31–50)	
	Post-op	25 (25–50)	25 (25–50)	25 (25–50)	
	Change	0 (−25 to 0)	−25 (−50 to 0)	0 (−25 to 0)	0.3 ^†^
	*p*-value *	<0.001	0.001	<0.001	
**Incontinence Impact**					
	Pre-op	67 (67–100)	100 (67–100)	67 (67–100)	
	Post-op	33 (33–42)	33 (33–67)	33 (33–67)	
	Change	−33 (−67 to 0)	−33 (−67 to 0)	−33 (−67 to 0)	0.8 ^†^
	*p*-value *	<0.001	<0.001	<0.001	
**Role Limitations**					
	Pre-op	67 (50–83)	67 (50–83)	67 (50–83)	
	Post-op	25 (0–33)	33 (33–50)	33 (0–50)	
	Change	−33 (−67 to 0)	−33 (−50 to 0)	−33 (−67 to 0)	0.8 ^†^
	*p*-value *	<0.001	<0.001	<0.001	
**Physical Limitations**					
	Pre-op	67 (50–83)	83 (50–92)	67 (50–83)	
	Post-op	17 (17–33)	33 (0–50)	17 (17–33)	
	Change	−33 (−67 to 0)	−33 (−75 to −8)	−33 (−67 to 0)	0.4 ^†^
	*p*-value *	<0.001	0.008	<0.001	
**Social Limitations**					
	Pre-op	33 (22–56)	33 (11–67)	33 (22–56)	
	Post-op	0 (0–11)	0 (0–33)	0 (0–11)	
	Change	−28 (−33 to 0)	−22 (−44 to 0)	−22 (−33 to 0)	0.7 ^†^
	*p*-value *	<0.001	0.04	<0.001	
**Personal Relationships**					
	Pre-op	33 (25–75)	50 (0–67)	42 (17–67)	
	Post-op	0 (0–33)	0 (0–42)	0 (0–33)	
	Change	−33 (−50 to 0)	0 (−42 to 0)	−33 (−50 to 0)	0.4 ^†^
	*p*-value *	<0.001	0.3	<0.001	
**Emotions**					
	Pre-op	33 (22–67)	44 (11–78)	33 (22–67)	
	Post-op	0 (0–11)	0 (0–33)	0 (0–22)	
	Change	−33 (−36 to 0)	−11 (−44 to 0)	−22 (−44 to 0)	0.5 ^†^
	*p*-value *	<0.001	0.07	<0.001	
**Sleep/Energy**					
	Pre-op	33 (33–67)	33 (17–50)	33 (33–62)	
	Post-op	17 (0–33)	17 (0–33)	17 (0–33)	
	Change	−25 (−33 to 0)	−25 (−33 to 0)	−25 (−33 to 0)	0.7 ^†^
	*p*-value *	<0.001	0.06	<0.001	
**Severity Measures**					
	Pre-op	67 (40–73)	73 (53–87)	67 (40–80)	
	Post-op	40 (20–60)	60 (27–67)	40 (27–67)	
	Change	−7 (−33 to 0)	−7 (−35 to 0)	−7 (−33 to 0)	1.0 ^†^
	*p*-value *	0.002	0.1	0.001	
**Overactive Bladder**					
	Pre-op	15 (11–17)	15 (11–19)	15 (11–17)	
	Post-op	7 (4–10)	8 (2–12)	8 (3–11)	
	Change	−6 (−11 to −1)	−7 (−9 to −5)	−6 (−11 to −1)	0.6 ^†^
	*p*-value *	<0.001	0.008	<0.001	
**Symptom Severity Scale**					
	Pre-op	62 (50–88)	81 (50–88)	62 (50–88)	
	Post-op	33 (0–56)	12 (0–62)	29 (0–62)	
	Change	−17 (−56 to 0)	−44 (−75 to −9)	−25 (−65 to 0)	0.2 ^†^
	*p*-value *	<0.001	0.006	<0.001	

Data are presented as median (25th–75th percentile). Scores range from 0–100, with higher scores indicating poorer quality of life. LSC = laparoscopic sacropexy; SSLF = sacrospinous ligament fixation. * Wilcoxon signed-rank test for paired comparisons (pre- vs. post-operative within group). ^†^ Mann–Whitney U test for between-group comparison of change scores.

**Table 5:** Patient satisfaction summary.

Satisfaction Measure	LSC (n = 39)	SSLF (n = 15)	Total (n = 54)	*p*-Value
Positive rating of treatment quality	36 (92.3)	13 (86.7)	49 (90.7)	0.32
Received desired treatment	37 (94.9)	14 (93.3)	51 (94.4)	0.62
Clinic met needs	37 (94.9)	13 (86.7)	50 (92.6)	0.32
Would recommend to friend	37 (94.9)	14 (93.3)	51 (94.4)	1.0
Satisfied with extent of help	37 (94.9)	13 (86.7)	50 (92.6)	0.32
Treatment helped cope with problems	34 (87.2)	14 (93.3)	48 (88.9)	1.0
Overall satisfaction	37 (94.9)	13 (86.7)	50 (92.6)	0.32
Would return for care if needed	37 (94.9)	15 (100)	52 (96.3)	1.0

Data are presented as number (percentage) of patients with positive responses. Positive responses include the following: Excellent/Good, Yes definitely/Generally yes, Very/Largely satisfied. LSC = laparoscopic sacropexy; SSLF = sacrospinous ligament fixation. No statistically significant differences were observed between groups (Fisher’s exact test).

## Data Availability

The data presented in this study are available on request from the corresponding author due to privacy restrictions.

## Abbreviations

The following abbreviations are used in this manuscript:

ASA	American Society of Anesthesiologists
BMI	Body mass index
FSFI	Female Sexual Function Index
IQR	Interquartile range
KHQ	King’s Health Questionnaire
LASH	Laparoscopic supracervical hysterectomy
LLS	Laparoscopic lateral suspension
LSC	Laparoscopic sacropexy
MCID	Minimally Clinically Important Difference
PFD	Pelvic floor dysfunction
POP	Pelvic organ prolapse
SSLF	Sacrospinous ligament fixation
SVD	Spontaneous vaginal delivery
